# Inconsistencies in Self-Reporting of Sexual Activity Among Young People in Nairobi, Kenya

**DOI:** 10.1016/j.jadohealth.2009.03.014

**Published:** 2009-12

**Authors:** Donatien Beguy, Caroline W. Kabiru, Evangeline N. Nderu, Moses W. Ngware

**Affiliations:** African Population and Health Research Center, Nairobi, Kenya

**Keywords:** Adolescents, Sexual activity, Inconsistency, Slum, Informal settlements, Nairobi, Kenya

## Abstract

**Purpose:**

Accurate and reliable data on the prevalence of adolescents' sexual behavior are paramount for effective sexual and reproductive health intervention. Adolescents' sexual behavior has been widely studied. However, scholars have raised concerns about the accuracy and reliability of self-reported sexual behavior by adolescents. Previous research shows high levels of adolescent sexual activity in urban informal settlements; yet, the accuracy of self-reported sexual experience in these settings is understudied.

**Methods:**

The objective of this article is to assess consistency of self-reported sexual activity among 2324 adolescents living in slum and nonslum settlements in Nairobi, Kenya. We examine two forms of inconsistencies, namely, what we term “reborn virgins” and inconsistent timing of sexual debut, during two rounds of survey. Factors influencing inconsistent reporting are explored through logistic regression.

**Results:**

A total of 469 (20%) adolescents gave inconsistent information on whether they have ever had sex (n = 190) or timing of first intercourse (n = 279). Males, slum residents, and adolescents attending school were more likely to give inconsistent sexual information. Among inconsistent reporters, slum residents, adolescents reporting substance use, and those with secondary (vs. primary) education were more likely to reclaim virginity status than to misreport the timing of first sex. However, older adolescents were less likely to reclaim virginity status.

**Conclusions:**

We found significant differences between adolescents who provide consistent reports and those who misreport sexual behavior data. We argue that researchers should account for biases stemming from misreporting of sensitive information among young people and, in particular, should be cognizant of how reporting quality may vary across demographic groups.

The validity and reliability of survey data collected on sensitive, illegal, and risk behaviors have been questioned by social science researchers in both developing and developed countries [1–4]. In sub-Saharan Africa, where high human immunodeficiency virus (HIV) prevalence rates and poor sexual and reproductive health outcomes have fueled the need to collect sexual behavior data, it has been indicated that the reporting of premarital sexual experience is of poor quality in many surveys [5,6]. It is widely recognized that disclosure of sexual activity is influenced by a variety of factors including sociocultural norms that set limits on acceptable sexual conduct [7], widespread policies advocating abstinence [8], and the promotion of premarital abstinence by influential conservative religious groups [6]. Other possible reasons for inconsistencies in sexual behavior data include recall bias stemming from the retrospective nature of most surveys, poor comprehension of survey questions, or low motivation for participation [2,7].

Since self-reported sexual behavior is difficult to validate, researchers have relied on checks of: the consistency of sexual data reported by individuals across time points [1,2,9], within-survey consistency of responses [10], or consistency between biological markers and self-reported data [11] .

Previous research has highlighted several differences between adolescents who provide consistent reports and those who do not. In their analyses of data from Demographic and Health Surveys in six sub-Saharan African countries, Zaba et al [5] reported inconsistencies in reported age at first sex among birth cohorts across time that were suggestive of young men exaggerating their sexual debut and young women denying their sexual activity. In Jamaica, Eggleston et al found that boys were more likely than girls to misreport sexual behavior [9]. In South Africa, nearly 40% of sexually active high school students who were found to inconsistently report their sexual experience were also more likely to misreport their substance use [2]. In the United States, Upchurch et al [3] reported that about 10% of adolescents enrolled in grades 7–12 reclaimed virginity status, with boys being more likely than girls to do so, whereas older adolescents, those not living with parents, and those having highly educated mother were less likely to reclaim initial report of sexual experience. Another study conducted in the United States showed that approximately one-third of adolescents gave inconsistent reports of sexual activity or age at intercourse. Race/ethnicity, gender, and socioeconomic status were found to be significantly associated with inconsistent report, with white females and those living in a two-parent family being the least likely to be inconsistent [1].

Many studies have focused on adolescents' sexual behavior in slum communities and highlighted riskier sexual practices among young people living in these resource-poor settings [12–15]. However, little attention has been paid to the accuracy and the reliability of the information gathered on the sexual activity of the young people in poor urban settings in developing countries.

The objective of this paper is to assess inconsistency of self-reported sexual activity among adolescents in slum and nonslum settlements in Nairobi, Kenya. The United Nations Human Settlements Programme (UN-HABITAT) defines a slum household as “a group of individuals living under the same roof that lack one or more of the following conditions: access to safe water; access to sanitation; secure tenure; durability of housing; and sufficient living area” [16]. In Kenya, approximately 71% of all urban dwellers are estimated to live in areas that meet the UN-HABITAT's definition of slums [16]. These slums are characterized by extreme poverty, poor sanitation, inadequate social services, insecurity, social fragmentation, and poor livelihood opportunities. Nairobi, the capital city, is home to several slums, where between 1 and 2 million people live in cramped conditions without proper access to sanitation or affordable clean water [15].

We examine two forms of inconsistencies, “reborn virgins” (i.e., report sexual experience during one wave of data and report no prior sexual experience at a later survey) and inconsistent timing of sexual debut (inconsistencies in age at first intercourse). We argue that these two types of inconsistencies are distinctly different and that they are likely to be influenced by different factors, thereby leading to different implications with respect to research and policy actions. Respondents who reclaim virginity at Wave 2 could have done so deliberately, showing low motivation or unwillingness to answer the survey questions; those who provide inconsistent information on the timing may have done so because of memory lapses.

## Methods

### Study design and procedures

The data analyzed in this paper are drawn from two waves of the Education Research Program (ERP), a longitudinal population-based study in two slum and two nonslum settlements in Nairobi, Kenya, that was designed to compare educational outcomes between slum and nonslum communities. The study is nested in the Nairobi Urban Health and Demographic Surveillance System (NUHDSS), which collects routine health and demographic data from more than 50,000 individuals living in more than 20,000 households in two slum areas in the city (Korogocho and Viwandani). The ERP has been following children and adolescents aged 5–19 years in these two slums and in two nonslum but low-income communities (Harambee and Jericho) since 2005. Wave 1 was conducted between June and September 2005, and Wave 2 was carried out between August and October 2006. Information on adolescent sexual and other risk behavior is collected as part of a child module questionnaire that is updated annually [17]. The behavior section of the child module is completed by adolescents at least 12 years of age. Ethical approval was granted by the Ethical Review Committee of the Kenya Medical Research Institute. Adolescents were briefed on their rights as participants in the study and provided verbal consent. Parental consent was also sought for all adolescents aged 12–17 years.

### Sample description

A total of 3936 adolescents aged 12–19 years completed the child module questionnaire in Wave 1. Of these, 2,324 adolescents (50% female and 50% male) from 1,655 households completed the second wave of data 1 year later and comprise the sample for this study. Adolescents who were lost to follow-up were more likely to be older. Males and adolescents who were in school during the first wave of data collection were less likely to be lost to follow-up. Adolescents who completed surveys in both waves did not differ significantly from those lost to follow-up based on slum residence or sexual experience.

A total of 469 adolescents (20%) gave inconsistent information on whether they had ever had sex (n = 190) or the timing of first intercourse (n = 279). Although we present descriptive information on the 2,324 respondents who completed both waves of surveys, we limit the bivariate and multivariate analyses to respondents who reported sexual activity in at least one wave (n = 966), as inconsistent reporting of sexual activity is possible only for this group. Thus, among the subsample of sexually active adolescents, 49% gave inconsistent information.

### Measures

Two outcome variables based on inconsistent reporting of sexual behavior were generated: what we term “reborn virgins” (i.e., adolescents who retracted a Wave 1 report of sexual activity in Wave 2), and inconsistent timing of sexual debut. At both waves respondents were asked, “Have you ever had sex?” and “How old were you when you first had sex?” Possible responses to the first question were “yes” or “no.” The second question was asked only when the response to the first was affirmative. Respondents who answered “yes” to the first question in Wave 1 and “no” to the same question in Wave 2 were coded as “reborn virgins.” Respondents who answered “no” to the first question in Wave 1 and “yes” to the same question in Wave 2 but reported a younger age at first intercourse than age at Wave 1 were coded as providing an inconsistent report of the timing of sexual debut. Respondents who made the transition to first sex between the two waves and gave the same age at first sex as age at Wave 1 or reported an older age of first sex than age at Wave 1 were coded as consistent reporters.

### Explanatory variables

We included as explanatory variables several sociodemographic factors that have been previously associated with sexual behavior: slum versus nonslum residence [12–14], gender [18], age [1,9], school attendance (current attendance and highest level of education) [19,20], and living arrangements (living with parents and number of adolescents in the household) [18,19,21]. School attendance and educational attainment are used as a proxy of literacy and may capture the ability of respondents to understand the wording of survey questions. We also included substance use (ever drank alcohol, and ever smoked cigarettes or used other drugs) as an explanatory variable, as researchers have found that early sexual debut is associated with risky behaviors including alcohol and drug use, with adolescents reporting alcohol and drug use being more likely to engage in sex [22–24]. We used explanatory variables measured in Wave 1.

Logistic regression models were used to examine factors associated with inconsistent reports of sexual behavior. The first set of logistic models was used to assess the factors associated with inconsistent reporting, irrespective of the type of inconsistency (inconsistent vs. consistent). To capture the factors explaining each type of reporting, a second set of logistic models was run to compare adolescents who retracted claims of sexual activity and those giving inconsistent information on the timing of first sex (reborn virgin vs. false timing). Only respondents with inconsistent reports are considered for the latter. The logistic regression estimates of the standard errors were adjusted for potential lack of independence among the adolescents within the same household using clustered sandwich estimator (Huber/White/sandwich estimate of variance) implemented in Stata 10 [25].

## Results

Table 1 summarizes demographic characteristics of respondents according to four categories of respondents: reborn virgins, inconsistent timing, consistent report of ever having had sexual activity (consistently/ever), and consistent report of never having had sexual activity (consistently/never). Respondents who consistently reported never having had sex were younger than the other groups (mean age of 13.9 years compared with 16.2–16.6 years for other groups). A greater proportion of respondents who retracted their initial report of sexual activity were slum residents (89%), compared with 74–78% among the other groups. Likewise, a greater proportion of respondents who retracted an initial claim of sexual activity had ever tried to smoke or used recreational drugs (33% vs. 5–21% for other groups) or had ever drunk alcohol (27% vs. 4–21% for other groups).

Several logistic models were run to examine possible factors that influence the odds of giving inconsistent reports of first sexual intercourse. First, we run separate models to capture the effect of each variable on the outcome variable (Table 2). Results shown in Table 2 indicate that only gender, schooling status, and number of adolescents living in the household were significantly associated with inconsistent reporting in the bivariate models. Male adolescents were approximately 1.4 times more likely to give inconsistent reports than their female counterparts. Adolescents who were in school at Wave 1 were also more likely to inconsistently report their first sex. Second, we run a full model including all the explanatory variables, allowing us to estimate the net effect of each variable. Results are also displayed in Table 2 (in the “Multivariate model” column). Gender, schooling status, and number of adolescents in the household remain significantly associated with inconsistency after adjusting for other factors in the multivariate logistic model. After adjusting for other factors in the model, slum residence and increasing age were also associated with higher chances of inconsistent reporting of sexual behavior, though the odds ratios were only marginally significant at the 10% level of significance. Third, we examined the chances of retracting an initial claim of sexual activity versus providing contradictory information on the timing of sexual debut. Only respondents who gave inconsistent reports are considered in this analysis. The chances of retracting an earlier claim of sexual activity compared with giving inconsistent information on the timing of first intercourse are thus estimated. Table 3 displays results of the regression analysis. Slum residents were 4.8 times more likely to retract previous reports of sexual activity than to provide inconsistent reports of timing of first sex. Older respondents were significantly less likely to retract previous report of sexual activity. Each additional year decreases by 20%, the chances of retracting a previous report of sexual activity rather than of giving inconsistent age at sexual debut. Respondents reporting substance use were significantly more likely to retract earlier reports of sexual activity. Adolescents with smoking and alcohol use experience were 2.8 and 3.0 times more likely to retract initial claim of sexual activity than to provide inconsistent reports of the timing of first sex. Having a secondary-level of education was associated with higher chances of reclaiming virginity status than that of giving contradictory information on the timing of sexual debut. Gender, schooling status, number of adolescents in the household, and family structure are not significantly associated with the chances of retracting earlier sexual experience.

## Discussion

The validity and reliability of sexual behavior data are critical in addressing sexual and reproductive health concerns of young people and in informing successful targeting and evaluation of sexual and reproductive health interventions. Understanding factors associated with inconsistent reporting of sexual behavior is therefore of prime importance in establishing the extent to which data that inform programmatic efforts can be relied upon. From a researcher's perspective, inconsistent information also presents a number of challenges in data analysis. The researcher may have to drop cases with inconsistent data and, in instances in which these cases comprise a large proportion of the data set, the effective sample size is greatly reduced. Furthermore, if the respondents providing inconsistent information belong to a group with unique characteristics, dropping them leads to selection bias.

Approximately 50% of sexually active adolescents in the sample provided inconsistent information on their sexual behavior. This proportion is large in light of other studies. In South Africa, Palen et al [2] found that approximately 40% of sexually active adolescents misreported their lifetime sexual experience. This proportion is also much higher than the proportion of respondents (37%) providing inconsistent reports of sexual activity in the study by Eggleston et al [9] among Jamaican adolescents. Of those giving inconsistent sexual information, 59% gave contradictory responses on the timing of first sex. Adolescent sexual activity generally occurs in a sociocultural context that generally disapproves of sex outside of marriage [26,27]. Thus, contradictory information on the timing of first sex may reflect possible discomfort in disclosing sexual activity during first interview.

As in the Jamaican study conducted by Eggleston et al [9], we found that, compared with girls, boys had a greater probability of misreporting their sexual experience, irrespective of the type of inconsistent reporting. Gender differences in sexual activity should therefore be discussed in light of the possible tendency by boys to provide inconsistent reports. In particular, researchers, policy makers and program designers should keep in mind these limitations when it comes to designing and evaluating programs or interventions targeting sexually active adolescents. Gender differences may be explained by the fact that in many societies, boys' sexual activity is often seen as an act of pride/honor, whereas girls' sexual experience could be interpreted as an act of social deviance. Accordingly, boys may feel considerable pressure to be sexually active. In a recent study examining Nigerian male youths' view on abstinence, Izugbara [28] reported that several participants highlighted the frustrations of being abstinent among sexually active peers. As one 21-year-old participant in that study put it, “All my friends were having sex and telling me about their experiences … I often felt very frustrated and unhappy” [28]. Furthermore, with increasing age and physical maturation, there may be more “freedom” to make a decision about one's sexual behavior.

Older adolescents were more likely to give inconsistent reports of sexual behavior than were younger adolescents. Moreover, older adolescents were less likely to retract previous claim of sexual activity than to inconsistently report the timing of first sex. We posit that with increasing age and physical maturation, sexual activity becomes normative; thus older adolescents may be less likely to retract an initial claim of sexual intercourse. However, the greater likelihood of misreporting the timing of first sex may stem from memory lapses [1] and from greater proneness to inconsistency in older adolescents compared with younger adolescents, because older adolescents are at the age when transition to first sex is likely to occur.

Our empirical findings showed that inconsistent reporters who indicate drug and alcohol use were more likely to reclaim virginity status than to provide inconsistent report of timing of sexual debut. However, we did not find a significant association between overall inconsistent report of sex and substance use. In general, researchers have found that adolescents reporting alcohol and drug use are more likely to engage in sex [22–24]. As Johnston [22] states, adolescents experiment with drugs and alcohol for various reasons including exposure to expanded opportunities and increased temptations, a widened social network as they grow older, as well as social pressure. These aspects of the social milieu may also present external pressure to be perceived to be sexually active. Other studies suggest that substance use and inconsistent reporting of sex may be influenced by similar factors, including embarrassment or stigma [2]. It seems that among our study participants, this explanation holds true only for the “retracting” type of inconsistency.

The multivariate analysis indicated a significant association between school attendance (Wave 1) and inconsistent reporting, with youth in school being more likely to misreport their sexual experience than youth not in school. Schools are often targeted sites for sexual and reproductive health programs that encourage abstinence; thus, students may be less inclined to correctly disclose their sexual experience. On the other hand, one could expect students to be able to provide more consistent answers on timing because of higher literacy levels. Palen et al [2] found that school failure as a proxy of low literacy did not significantly influence inconsistent reporting of sex among South African adolescents. Although school attendance was not significantly associated with the type of inconsistency, our results show that compared with adolescents with a primary level of education, those with a secondary level of education are more likely to retract their initial claim of sexual activity than to misreport the timing of sexual debut. These findings are consistent with the assumption that better educated adolescents have higher literacy levels and are more likely to provide valid answers related to timing. These findings also suggest that inconsistent reporting is unlikely to be induced by a misunderstanding or poor incomprehension of the survey questions. Instead, better educated adolescents may be unwilling to disclose sexual activity, given that schools are often the settings for sexual and reproductive health interventions that often advocate abstinence.

Several limitations should be kept in mind when interpreting the findings of this study. First, as pointed out by Palen et al [2] although we state that there are inconsistencies in reporting between waves, we do not know where the inconsistencies lie. In other words, we do not know which report (initial or subsequent or both) is misrepresented. We are also unable to ascertain whether consistent information given by some adolescents was accurate. Second, we did not assess the possible effect of other factors on the respondents' answers, including the interviewers' characteristics and more broadly the conditions of the interviews. Third, our sample is limited to those who provided data in both waves. This may introduce a selection bias; however, we note that adolescents who were lost to follow-up did not differ from those with complete data on the primary variable of interest, namely, self-reported sexual activity.

Despite these limitations, our findings have implications for researchers and end users of research. Researchers should account for biases stemming from misreporting of sensitive information among young people and, in particular, should be cognizant of how reporting quality may vary across demographic groups. Younger respondents may be inclined to withhold information about behavior considered to be inappropriate if they are being interviewed by adults. Furthermore, the level of privacy in which an interview of such sensitive nature is performed may also affect how truthful or consistent respondents may be. In this case, the level of privacy in the slum may be low because of close proximity of structures and walls made of flimsy material, such as mud or cardboard.

Alternative forms of interviewing may be necessary. For instance, Turner et al [29] in a study conducted in the United States found that respondents using an audio computer-assisted self-interviewing (ACASI) device gave more consistent sexual behavior data than those who completed either a written questionnaire or an oral interview. However, the effectiveness of ACASI in the sub-Saharan Africa context is unclear. For example, in Nyeri District in Kenya, Mensch et al [6] reported that, by comparison with face-to-face interviews, ACASI did not improve the reporting of sexual activity among young girls. Likewise, in their study in Kisumu District in Kenya, Hewett et al [10] reported that compared with the computerized interviews, self-administered interviews produced highly consistent reports of sexual activity among adolescents girls. These findings led the authors to conclude that the privacy provided by the ACASI mode could be overshadowed by the respondents' attitudes toward the computer. Further research on methodologies to improve the quality of data collected and to address these inconsistencies is also needed. This kind of research could be built on the experiences of other studies [6,10,30, 31] that aim to improve the quality of answers to sensitive survey questions.

## Figures and Tables

**Table 1:** Percentage distribution of adolescents by background characteristics and inconsistencies

Characteristics	Sexually experienced			Not sexually experienced		Inconsistent among sexually active
	“Reborn virgin”	Inconsistent timing	Consistent ever	Consistent never	Total	
Mean age (SD) years	16.2 (1.7)	16.6 (1.8)	16.5 (1.9)	13.9 (1.8)	14.9 (2.2)	16.5 (1.8)
Slum resident	89.0	75.6	74.1	77.7	76.3	49.6
Female	37.9	47.3	51.3	51.9	50.1	44.4
Ever smoked or used drugs	33.2	11.8	20.7	4.8	11.4	48.2
Ever consumed alcohol	26.8	10.8	21.3	3.7	10.2	43.3
In school at Wave 1	70.0	68.1	56.5	94.1	81.0	53.5
Highest education level∗						
Primary	56.8	56.6	59.0	75.1	67.9	47.6
Secondary	31.6	32.6	30.0	15.9	22.2	50.3
Living arrangements∗						
Two-parent family	34.2	28.3	32.4	38.1	35.4	47.2
One-parent family	18.4	21.9	19.7	18.4	19.1	49.5
Other	33.2	42.3	39.2	31.3	34.5	48.1
No. of adolescents in household∗						
One	17.9	21.5	21.9	23.3	22.3	46.3
Two	26.8	31.2	33.2	29.4	30.2	45.5
Three	23.7	24.7	17.3	19.7	20.1	57.0
Four or more	17.4	15.1	18.9	15.5	16.3	44.4
n	190	279	497	1358	2324	966
%	8.2	12.0	21.4	58.4	100.0	48.6

∗ For education, living arrangements, and number of adolescents in the household, the total percentage does not add up to 100 because of missing values. Missing values are included in the logistic models to reduce bias stemming from deletion of missing values.

**Table 2:** Odds ratios (OR) and robust 95% confidence intervals (95% CI) for predictors of inconsistent reporting of sexual activity (logistic regression)

Variable	OR (95% CI)	
	Bivariate model	Multivariate model
Slum resident (ref: nonslum resident)	1.2 (.9–1.7)	1.5∗ (1.0–2.3)
Male (ref: female)	1.4∗∗ (1.1–1.8)	1.4∗∗ (1.0–1.8)
Age	1.0 (.9–1.1)	1.1∗ (1.0–1.2)
Tried smoking or used drug	1.0 (.7–1.4)	1.0 (.7–1.5)
Ever consumed alcohol	.8 (.6–1.1)	.7 (.5–1.1)
Attending school (Wave 1)	1.7∗∗∗ (1.3–2.2)	1.9∗∗∗ (1.4–2.6).
Education level (ref. primary)		
Secondary	1.1 (.8–1.5)	1.1 (.8–1.6)
Living arrangements (ref. one-parent family)		
Two-parent family	1.0 (.7–1.5)	1.0 (.7–1.4)
Other	1.0 (.7–1.3)	.8 (.6–1.1)
Number of adolescents in household (ref: one)		
Two	1.0 (.7–1.4)	.9 (.7–1.4)
Three	1.5∗∗ (1.0–2.3)	1.6∗∗ (1.0–2.4)
Four or more	.9 (.6–1.4)	.9 (.6–1.4)
Observations	–	966
Wald χ^2^	–	39.26∗∗∗
Log likelihood	–	−648.52

Ref. = reference.

∗ *p* < .1.

∗∗ *p* < .05.

∗∗∗ *p* < .01.

**Table 3:** Odds ratios (OR) and robust 95% confidence intervals (95% CI) for predictors of type of inconsistency (“reborn virgin” vs. inconsistent timing) of sexual activity (logistic regression)

Variable	OR (95% CI)	
	Bivariate model	Multivariate model
Slum resident (ref: nonslum resident)	2.6∗∗∗ (1.5–4.5)	4.8∗∗∗ (2.1–10.7)
Male (ref: female)	1.5∗ (1.0–2.2)	1.1 (.7–1.7)
Age	.9∗∗ (.8–1.0)	.8∗∗ (.7–1.0)
Tried smoking or used drug	3.7∗∗∗ (2.3–6.0)	2.8∗∗∗ (1.6–4.9)
Ever consumed alcohol	3.0∗∗∗ (1.8–5.1)	3.0∗∗∗ (1.6–5.7)
Currently attending school (Wave 1)	1.1 (.7–1.6)	.9 (.5–1.5)
Education level (ref. primary)		
Secondary	1.0 (.6–1.5)	2.1∗∗ (1.1–4.0)
Living arrangements (ref: one-parent family)		
Two-parent family	1.1 (.6–1.8)	.7 (.4–1.3)
Other	1.5 (.9–2.5)	1.1 (.6–1.9)
Number of adolescents in household (ref: one)		
Two	1.0 (.6–1.8)	.8 (.5–1.5)
Three	1.2 (.6–2.1)	1.1 (.6–2.2)
Four or more	1.4 (.7–2.7)	1.3 (.6–2.6)
Observations	–	469
Wald χ^2^	–	58.40∗∗∗
Log likelihood	–	−278.20

∗ *p* < .1.

∗∗ *p*<.05.

∗∗∗ *p*<.01.
